# Botulinum neurotoxin–encoding plasmids can be conjugatively transferred to diverse clostridial strains

**DOI:** 10.1038/s41598-018-21342-9

**Published:** 2018-02-15

**Authors:** Erin M. Nawrocki, Marite Bradshaw, Eric A. Johnson

**Affiliations:** Department of Bacteriology, University of Wisconsin-Madison, Madison, Wisconsin USA

## Abstract

Most Group I *Clostridium botulinum* strains harbor botulinum neurotoxin (*bont*) genes on their chromosome, while some carry these genes (including *bont/a*, *bont/b*, and *bont/f*) on large plasmids. Prior work in our laboratory demonstrated that Group I BoNT plasmids were mobilized to *C. botulinum* recipient strains containing the Tn*916* transposon. Here, we show that Tn*916* is nonessential for plasmid transfer. Relying on an auxotrophic donor phenotype and a plasmid-borne selectable marker, we observed the transfer of pCLJ, a 270 kb plasmid harboring two *bont* genes, from its host strain to various clostridia. Transfer frequency was greatest to other Group I *C. botulinum* strains, but the plasmid was also transferred into traditionally nontoxigenic species, namely *C. sporogenes* and *C. butyricum*. Expression and toxicity of BoNT/A4 was confirmed in transconjugants by immunoblot and mouse bioassay. These data indicate that conjugation within the genus *Clostridium* can occur across physiological Groups of *C. botulinum*, supporting horizontal gene transfer via *bont*-bearing plasmids. The transfer of plasmids possessing *bont* genes to resistant *Clostridium* spp. such as *C. sporogenes* could impact biological safety for animals and humans. These plasmids may play an environmental role in initiating death in vertebrates, leading to decomposition and nutrient recycling of animal biomass.

## Introduction

*Clostridium* is a diverse genus whose members are gram-positive, spore-forming, anaerobic bacteria. The species *C. botulinum* is notable for its production of botulinum neurotoxins (BoNTs), zinc-dependent proteases that specifically cleave SNARE proteins, preventing the release of neurotransmitters from synaptic vesicles^1^. BoNTs attack “the most vulnerable synapses”^2^ including the hemidiaphragm, leading to respiratory arrest and death. BoNTs are categorized into seven serotypes, designated A through G on the basis of their neutralization against death in mice with homologous antisera. The diverse *Clostridium* strains expressing the various BoNT serotypes are classified into four physiological Groups, designated with numerals I-IV. Group I encompasses proteolytic strains producing BoNT/A, /B, and /F, which are responsible for the vast majority of human botulism cases in the USA^3^. Historically, BoNT formation and lethality testing in mice were the only criteria required for designation of the species *C. botulinum*, a framework that has resulted in the complicated genome-based phylogeny of the species today^4^. The availability of sequence information has revealed the extensive genetic distance between the four Groups.

The genomic sequences from BoNT-producing clostridia have also helped to discern important phenotypic properties. These include environmental, nutritional, and molecular mechanisms governing BoNT expression, proteolytic processing and activation, cell lysis and release of BoNT, and stability of BoNT in culture^5,6^. Genomic studies have also been important in revealing and understanding atypical neurotoxigenic strains, such as those that possess two (or three) BoNT gene clusters, express more than one BoNT in culture, produce chimeric BoNTs including BoNT/FA, or encode BoNT-like molecules in non-clostridial organisms^7–9^. Sequence comparisons indicate that *C. botulinum* strains and BoNTs have evolved independently of one another, suggesting that horizontal gene transfer may play a role in toxin acquisition^10–12^.

Further evidence for horizontal gene transfer in *C. botulinum* is given by the abundance of genes for mobile elements surrounding the BoNT gene clusters^13^. All *bont* genes are encoded as part of a conserved cluster, where they are found linked to genes encoding accessory or non-toxic complex proteins. In most sequenced strains, *bont* gene clusters are located in specific positions on the chromosome or plasmid. Analyses of the neighboring regions often identify nearby elements that may be involved in recombination or other functions, including flagellin genes and IS elements^14^. Intact or partial flagellin genes are present upstream of the cluster in some strains, and are frequently hypervariable sites of recombination in other species^15–18^. Both plasmid-borne and chromosomally located toxin gene clusters are flanked by mostly defective IS elements of various families. When intact, IS elements encode transposase enzymes that promote their own translocation. The IS elements identified near the toxin gene clusters in Group I strains have so far been shown to be genetically degraded, sharing a maximum of 83% amino acid homology with the full-length elements^19^. The recombination events that inserted toxin clusters into their modern loci are therefore assumed to have occurred in the distant past.

Weickert *et al*. detected plasmids in *C*. *botulinum* type A strains; however, strains cured of plasmids still produced BoNT^20^. The first report of plasmid-borne *bont* genes in Group I *C. botulinum* strains was made in 2007^21^, and was confirmed by Smith *et al*. in genomic sequencing studies^19^. Despite recent advances in clostridial plasmid biology, particularly in *Clostridium perfringens*^22^, the BoNT plasmids are poorly characterized. In Group I *C. botulinum* strains, they range in size from approximately 140–270 kb and encode BoNTs of serotypes A, B, F, or multiple BoNTs^19,23,24^. Although comparatively few completed sequences are available, the presence of *bont* genes on plasmids is likely widespread; one survey found *bont/b* on a plasmid in 32 of 60 strains examined, including 21 of 22 *bont/b1* strains^23^. A recent study extends this observation, showing similar plasmids harboring *bont*/*b6* present in strains of both the *C. botulinum* and *C. sporogenes* chromosomal lineages^12^. Multi-domain homologues of BoNTs were also newly identified in a separate class of Firmicutes, raising questions about the origin and distribution of the neurotoxin^7^. Expression of one such homolog, BoNT/Wo, reveals that the novel toxin possesses a metalloprotease domain similar to BoNT/B but cleaves VAMP at a unique site^8^. These discoveries, in addition to the well-documented prophage-borne BoNT/C and /D in Group III *C. botulinum*^25^ and plasmid-borne BoNT/G in Group IV^26^, strongly imply a role for horizontal gene transfer in shaping the modern species. Movement of BoNT-encoding plasmids into nontoxigenic clostridial strains, particularly species with more resistant phenotypes, would pose real threats to health and safety, especially given the wide prevalence of clostridia in the environment and in the mammalian gastrointestinal tract.

Despite their large natural reservoir, the functions of *C. botulinum* and BoNTs in the environment are unknown. *C. botulinum* spores are ubiquitous in soils, dust, and marine sediment, and the species can persist in the intestines of healthy animals^27^. When vertebrates die and their carcasses decompose, the associated microbes and their community dynamics can change dramatically, facilitating shifts in nutrient cycling and rapid ecological succession^28^. In communities like the intestinal and soil microbiomes, conjugation serves as an efficient way to transfer beneficial genes, as exemplified by widespread antibiotic resistance^29^. The acquisition and formation of BoNTs may give clostridia a selective advantage by triggering vertebrate death and decomposition, thus generating a nutrient-rich substrate for population growth and perpetuation of the species^30^.

Although long suggested^31^, the ability of BoNT plasmids and their toxin genes to be mobilized remained unclear for many years. It was not until 2010 that experiments by Marshall *et al*. first demonstrated the movement of three BoNT-encoding plasmids between strains of *C. botulinum*, with data suggesting conjugation as the mechanism^32^. In the initial study, the plasmid-borne and/or chromosomal genes required for transfer were not identified^32^. Furthermore, the recipient strains contained a copy of Tn*916*; this transposon was artificially introduced to *C. botulinum* strains from an enterococcal host in our laboratory and served as a suitable positive selection marker (Tc^R^) for transconjugants^32,33^. As Tn*916* is a conjugative transposon that can mobilize non-conjugative plasmids^34,35^, its involvement in plasmid mobilization could not be excluded in the earlier study^32^. The mobility of Tn*916* was also problematic on the rare occasions that the transposon excised from the recipient and integrated into the donor, conferring the same antibiotic resistance phenotype as plasmid conjugation (Em^R^Tc^R^)^32^. In order to further investigate whether BoNT-encoding plasmids are conjugative, this study has recapitulated plasmid transfer in the absence of Tn*916*. The work has additionally examined the transfer of a BoNT-encoding plasmid to a wider range of clostridial recipient strains than previously shown.

## Results

### MNNG mutagenesis generates stable *C. botulinum* auxotrophs

**Figure 1:**
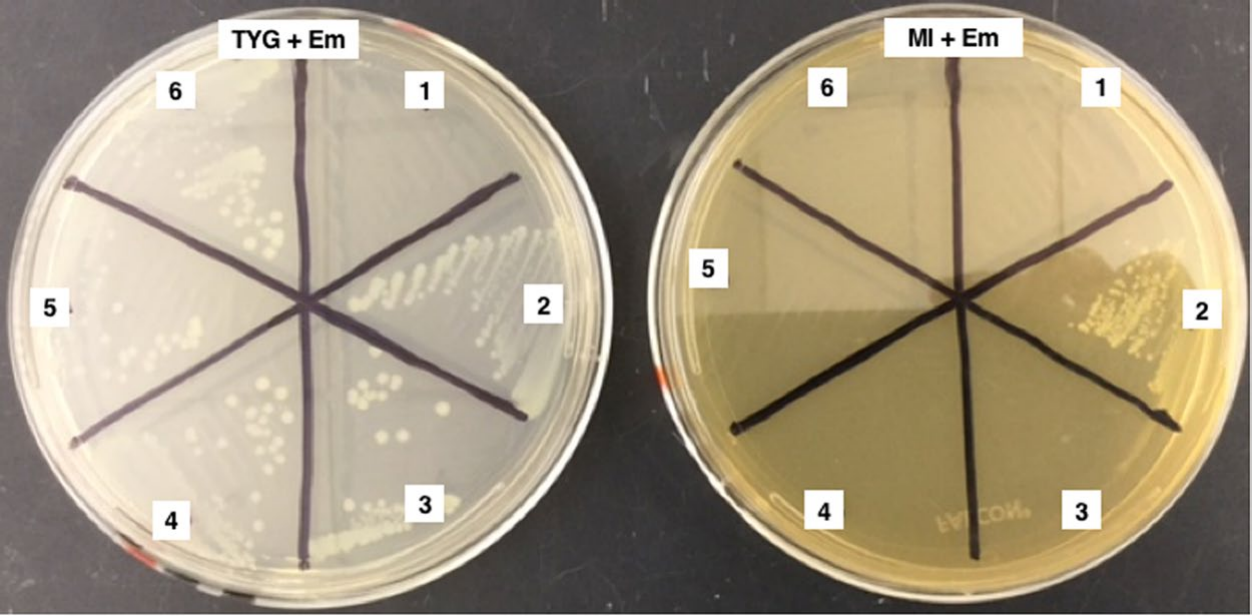
657Ba derivatives fail to grow on minimal medium. 1, *C. botulinum* 657Ba; 2, *C. botulinum* 657Ba (pCLJ-Erm); 3–6, EMN053–056. See Table 1 for additional strain information. At left, rich medium with 50 μg/ml erythromycin. At right, minimal medium with 50 μg/ml erythromycin. Auxotrophs can be restored to wild-type growth levels with the addition of 2 g/l lysine.

Prior experiments demonstrated the transfer of pCLJ to *C. botulinum* strains containing Tn*916*, a conjugative transposon that confers tetracycline resistance^32^. In order to eliminate the need for a second selective marker in the recipient strain and to expand the range of potential recipients, an auxotrophic donor strain was created. After multiple attempts to chemically mutagenize *C. botulinum* strain 657Ba (pCLJ-Erm) (Table 1) with *N*-methyl-*N*′-nitro-*N*-nitrosoguanidine (MNNG), a single lysine auxotroph was isolated from approximately 200 screened colonies. Lysine is nonessential for Group I *C. botulinum* and was chosen for this screen based on an auxotrophy identified in a prior mutagenesis experiment^33^. The current mutant has no apparent growth defects in rich medium; it grows on minimal medium (MI)^36^ supplemented with lysine but not on unsupplemented MI (Fig. 1). The auxotrophy is stable over repeated passages and reverts at low frequency (less than 10^−10^).

**Table 1:** Bacterial strains and oligonucleotide primers.

Name	Relevant Characteristics	Reference
*C. botulinum* 657Ba	*bont*/*b5* and *bont*/*a4* on pCLJ	^19^
*C. botulinum* 657Ba (pCLJ-Erm)	*bont*/*b5*::*ermB* and *bont*/*a4* on pCLJ	^32^
EMN053-EMN056	*bont*/*b5*::*ermB* and *bont/a4* on pCLJ, lys^−^	This study
*C. botulinum* Hall A-*hyper*	*bont*/*a1* on chromosome	^60^
*C. botulinum* Hall A-*hyper*/Tn*916*	Tc^R^, *bont*/*a1* on chromosome	^32^
*C. botulinum* 62A	*bont*/*a1* on chromosome	^61^
*C. botulinum* LNT01	Tc^R^, nontoxigenic	^62^
*C. botulinum* Marsh 51B	*bont*/*c* on prophage	EAJ*
*C. sporogenes* C	Nontoxigenic	EAJ*
*C. sporogenes* 4439	Nontoxigenic	EAJ*
*C. sporogenes* WR5	Nontoxigenic	EAJ*
*C. sporogenes* 19404	Nontoxigenic	^63^
*C. sporogenes* PA3679	Nontoxigenic	^64^
*C. butyricum* 13983	Nontoxigenic	EAJ*
*C. butyricum* 5520	*bont/e* on chromosome	^65^
A1-500F	GCTTTGGACATGAAGTTTTGAATC	This study
A1-1825R	GTTCTACCCAGCCTAAAAACATAG	This study
A4-535F	GGTTATGGTTCTACTCAATACATTAG	This study
A4-809R	CCCCCAAATGTTATAAGTTCCTC	This study
A4-1030F	CTGTTAACAGAGATTTACACAGAGG	This study
A4-1824R	CCAATTAACAAACGTAACTGCCTC	This study
A4-2001F	TGTACCAGAGATTGCGCTACCTG	This study
A4-2674R	CTATTAAATCATCATCTTTATATACTATGCTC	This study

All strains were grown under standard anaerobic culture conditions. Where possible, a source describing the isolation or characterization of the strain is given. Additional information is available upon request.

*Strain names given as in laboratory records. Provenance unknown.

### Plasmid transfer does not require Tn*916*

**Figure 2:**
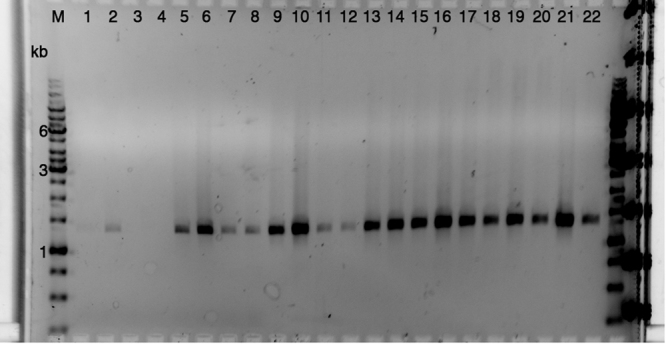
Colony PCR confirms transconjugant genotype. A marker gene characteristic of the recipient strain (*bont/a1*) was amplified by colony PCR using the A1 primer pair in Table 1 and analyzed by agarose gel electrophoresis. Lanes 1–2, recipient strains; lanes 3–4, pCLJ donor strains; lanes 5–22, selected transconjugants. M, O’GeneRuler 1 kb DNA ladder (Thermo Scientific, Waltham, MA). A full-length gel is presented in Supplementary Figure S1.

As the lysine auxotrophs retained the Em^R^ phenotype of the parent strain, they were used to demonstrate that pCLJ could be conjugated to unmarked recipients in a Tn*916*-free mating scheme (Table 1). Transfer frequencies for pCLJ ranged as high as 10^−6^ transconjugants per donor cell (Table 2). To confirm that the observed transconjugants were not revertant donor colonies, a subset was initially analyzed by PCR. One representative experiment is shown, in which 100% of putative transconjugants possessed the *bont*/*a1* gene indicative of the recipient background (Fig. 2). Certain unmarked recipient strains, namely Hall A-*hyper* and 62A, have Tn*916* derivatives generated for use in prior experiments in our laboratory. In order to evaluate the transposon’s role in plasmid transfer, these derivatives (strains Hall A-*hyper*/Tn*916* and LNT01, respectively) were used as recipients in selected auxotrophic mating experiments (Table 1). In these experiments, Tn*916* was present on the chromosome of recipients but was not used as a selectable marker. The transfer frequency of pCLJ to recipients carrying Tn*916* did not significantly differ from the transfer frequency to their parent strains, suggesting that the transposon was nonessential for plasmid mobilization (Table 2, Student’s *t*-test: Hall A-*hyper* vs. Hall A-*hyper*/Tn*916*, *P* = 0.15; 62A vs. LNT01, *P* = 0.1153).

**Table 2:** pCLJ is capable of conjugative transfer into a variety of clostridial strains.

Species	Strain	*C. botulinum* Group	BoNT Serotype	Mean Transfer Frequency
*C. botulinum*	Hall A-*hyper*	I	A	2.0 × 10^−6^ ± 2.8 × 10^−6^
	Hall A-*hyper*/Tn*916*	I	A	6.0 × 10^−6^ ± 6.9 × 10^−6^
	62A	I	A	3.9 × 10^−9^ ± 6.5 × 10^−9^
	LNT01	I	*	9.9 × 10^−11^ ± 1.6 × 10^−10^
	Marsh 51B	III	C	1.3 × 10^−13^ ± 1.5 × 10^−13^
*C. sporogenes*	C			1.1 × 10^−10^ ± 1.9 × 10^−10^
	4439			1.1 × 10^−10^ ± 1.9 × 10^−10^
	WR5			1.3 × 10^−10^ ± 2.1 × 10^−10^
	19404			1.5 × 10^−13^ ± 1.2 × 10^−13^
	PA3679			3.7 × 10^−14^ ± 4.8 × 10^−14^
*C. butyricum*	13983			1.4 × 10^−10^ ± 8.4 × 10^−11^
	5520	VI	E	2.4 × 10^−10^ ± 3.2 × 10^−10^

pCLJ, a 270 kb plasmid encoding two BoNTs, was transferred from *C. botulinum* 657Ba to selected recipient strains. Plasmid transfer was conducted in overnight matings on solid media. Transfer frequencies were calculated by dividing transconjugant CFU/ml by the larger of the donor or recipient CFU/ml. Minimum *n* = 3, ± s.d.

*Nontoxigenic Tn*916* mutant derived from strain 62A.

### BoNT-encoding plasmids are transferred to various clostridial species

A diverse panel of Group I *C. botulinum* strains received pCLJ in conjugative matings (Table 2). pCLJ was also capable of transfer into non-*botulinum* species. *C. sporogenes* recipients had lower transfer frequencies than *C. botulinum* recipients. *C. butyricum* strains were also suitable recipients, with transfer frequencies around 10^−10^ transconjugants per donor (Table 2). Although the incompatibility group of pCLJ is not known, the complete nucleotide sequence of *C. botulinum* strain 657Ba possesses two extrachromosomal replicons—the 270 kb plasmid described in this work, and a second cryptic plasmid of approximately 10 kb (NCBI Reference Sequence NC_012657.1). The smaller plasmid has not been purified or functionally characterized, nor have we monitored its stability or segregation in our laboratory isolates. Attempts to transfer pCLJ to a recipient strain with a comparably large plasmid were unsuccessful, as matings between the pCLJ donor and *C. botulinum* strain Okra B, which contains a BoNT-encoding plasmid of ~149 kb, did not yield transconjugant colonies. This suggests that the large plasmids of Group I *C. botulinum* are mutually exclusive, and available sequence information agrees that bivalent strains do not have more than one large BoNT-encoding plasmid^37^.

### pCLJ is stable in transconjugant strains and produces active BoNT/A4

**Figure 3:**
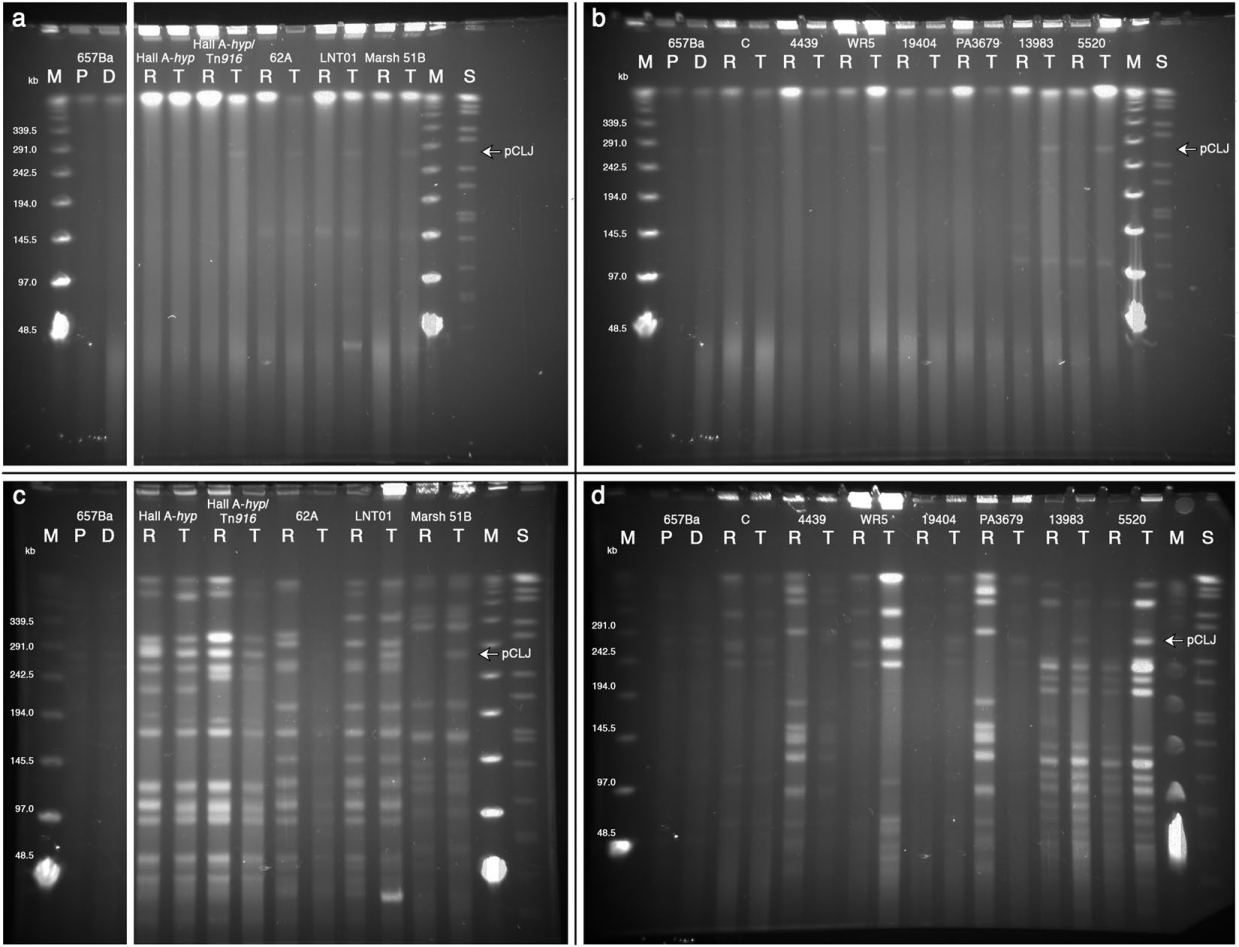
Full-length pCLJ is maintained in transconjugant strains. Matings were conducted as described in the text. PFGE plugs were prepared from the given strains and electrophoresed undigested (**a** and **b**) and following XhoI digest (**c** and **d**). pCLJ, at ~270 kb, is marked with an arrow. P, *C. botulinum* 657Ba (pCLJ-Erm) parent strain; D, pCLJ auxotrophic donor strain; R, recipient strains; T, transconjugant strains; M, Lambda PFG Ladder; S, *Salmonella enterica* serotype Braenderup strain H9812, XbaI digest^59^. All full-length gels are presented in Supplementary Figure S2.

**Figure 4:**
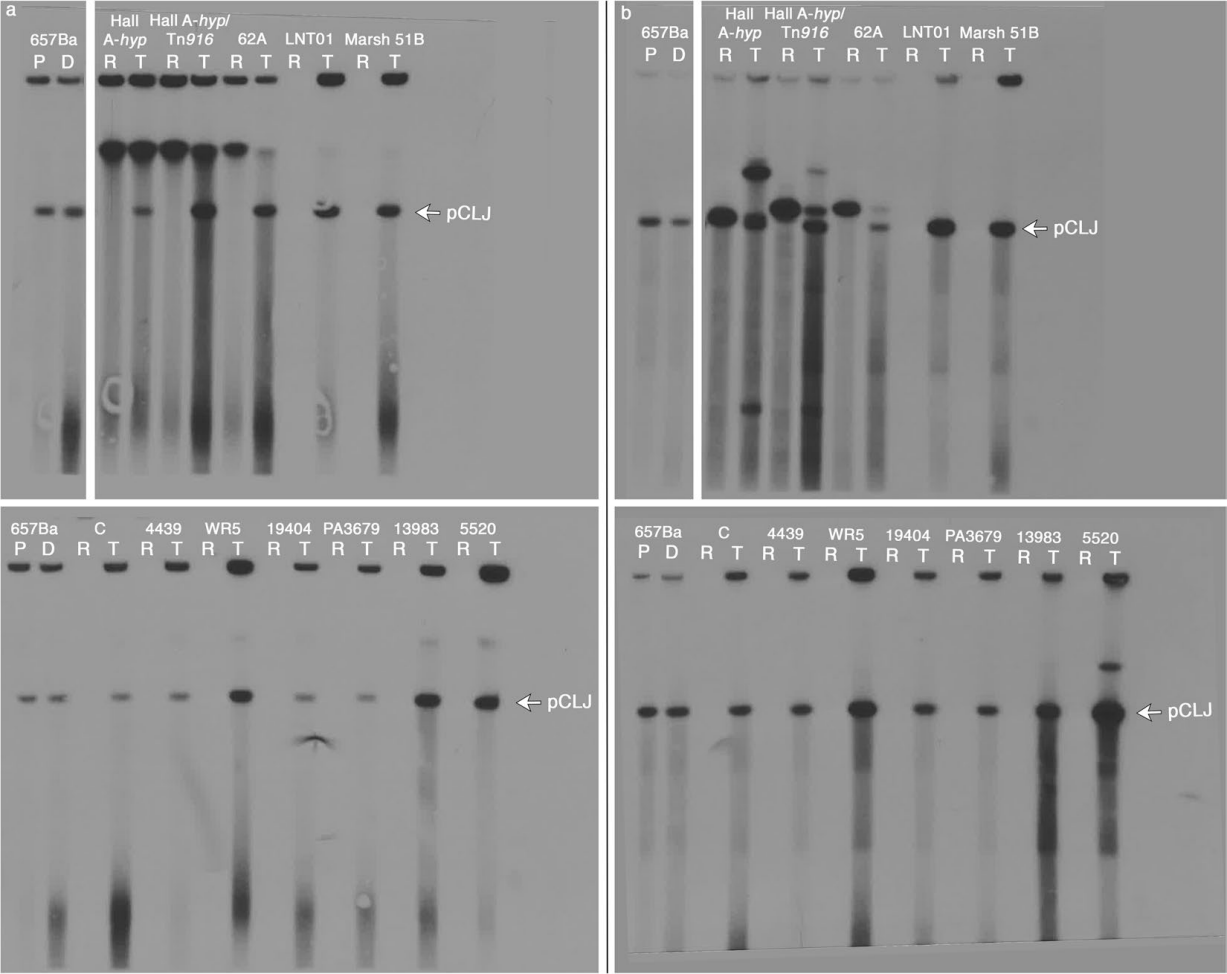
*bont/a4* remains associated with pCLJ in transconjugant strains. Undigested PFGE samples (**a**) and XhoI-digested samples (**b**) were transferred to nylon membranes and hybridized to *bont/a4* probes. pCLJ, at ~270 kb, is marked with an arrow. P, *C. botulinum* 657Ba (pCLJ-Erm) parent strain; D, pCLJ auxotrophic donor strain; R, recipient strains; T, transconjugant strains. Uncropped blots are presented in Supplementary Figure S3.

**Figure 5:**
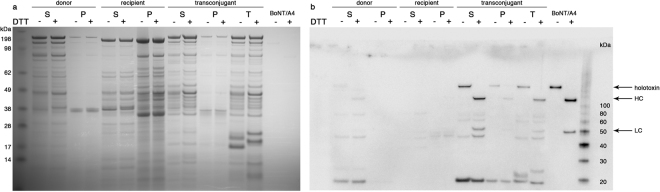
Transconjugant strains are converted to toxigenicity. Samples of donor strain EMN053, recipient strain *C. sporogenes* PA3679, and transconjugant strain *C. sporogenes* PA3679 (pCLJ) were collected at 48 h and prepared as described in the text. S, supernatant; P, pellet; T, trypsinized supernatant. Gels were (**a**) stained to visualize total proteins or (**b**) immunoblotted for the presence of BoNT/A. Purified BoNT/A4 was used as a control. Samples were reduced with 100 mM DTT as indicated. Full-length images are presented in Supplementary Figure S4.

As all transconjugants were recovered on the basis of plasmid-encoded antibiotic resistance, pCLJ was functional upon transfer to recipient strains. In the absence of antibiotic pressure and under standard culture conditions, the plasmid was maintained for at least five passages (data not shown). pCLJ was fully transferred to recipient strains, as evidenced by the appearance of a ~270 kb band in PFGE samples of representative transconjugants (Fig. 3). Moreover, the ~270 kb band consistently hybridized with radiolabeled probes against pCLJ’s active neurotoxin gene, *bont/a4* (Fig. 4). Note that the chromosomal *bont/a1* genes of certain transconjugant strains were also detected by the *bont/a4* probe; the high sequence identity between the two toxins prevented us from designing a truly plasmid-specific *bont/a* probe (Fig. 4). At the protein level, the cell lysates of parent and transconjugant strains had similar profiles, suggesting that the proteome was not globally altered by the presence of pCLJ (Fig. 5a). Plasmid genes were expressed in the transconjugant, as BoNT/A4 was detected in *C. sporogenes* (pCLJ) culture supernatants (Fig. 5b; Supplementary Figure S5). As determined by immunoblot at 48 h, the BoNT was primarily in the supernatant of cultures sampled, and it was proteolytically nicked as evidenced by the appearance of two bands following reduction of the disulfide bond (Fig. 5b). Culture supernatants of plasmid donor strains and *C. sporogenes* (pCLJ) transconjugant strains, but not of the nontoxigenic *C. sporogenes* PA3679 recipient strain, were lethal in the mouse bioassay. Mice were protected by neutralization with monovalent antibody against BoNT/A1 (Table 3).

**Table 3:** Transconjugants produce active BoNT/A4.

Sample	Treatment	Day 1	Day 2	Day 3
EMN053 (pCLJ donor)		2/2	2/2	0/2
*C. sporogenes* 4439 (pCLJ)		2/2	0/2	
*C. sporogenes* C (pCLJ)		2/2	0/2	
*C. sporogenes* PA3679		2/2	2/2	2/2
*C. sporogenes* PA3679 (pCLJ)		2/2	0/2	
*C. sporogenes* PA3679 (pCLJ)	Incubated with antitoxin	2/2	2/2	2/2

Two mice per sample were injected with culture supernatants (treated as described) and observed for signs of botulism. Numerators indicate mice surviving on a given day.

## Discussion

The functional and molecular aspects of BoNT-encoding plasmids, despite their likely role in the horizontal transfer of neurotoxin genes, remain largely enigmatic. Prior to this work, BoNT-encoding plasmids had been experimentally transferred, but only to *C. botulinum* recipients containing Tn*916*. Conjugation was the likely mechanism, as DNA transfer required cell-cell contact, was not mediated by donor culture supernatants, and was not prevented by DNaseI treatment^32^. Here, we have excluded Tn*916* from mating pairs to demonstrate that the BoNT-encoding plasmid pCLJ is not mobilized via transposon. In addition, we have extended the host range of pCLJ to non-*botulinum* species of *Clostridium*, supporting the hypothesis that horizontal gene transfer contributes to the diversity, toxigenicity, and function of the genus.

Transfer of BoNT plasmids is a low frequency event under our experimental conditions, and may well be even lower in natural environments. Nevertheless, given the extreme potency of BoNTs, their movement into new strains is cause for concern. Conjugation of BoNT plasmids into formerly nontoxigenic species poses threats to animal and human health and food safety, as these species have different disease tropisms and resistance phenotypes than *C. botulinum*. Strains producing BoNTs from the chromosome were typically amenable to plasmid transfer (Table 2). Recipient strains producing BoNT from their own plasmid, e.g. Okra B, did not yield appreciable pCLJ transconjugants, indicating that the toxin plasmids in Group I *C. botulinum* may be incompatible. It is possible that pCLJ is capable of conjugative transfer to plasmid-bearing strains outside Group I, but this was not determined due to the limitations on media and culture conditions in the current assay. Multiple plasmids of varying sizes coexist in Group III *C. botulinum* strains, but to our knowledge, only one plasmid lineage per cell carries the *bont* gene^25,38^. In species that are already toxigenic, the introduction of a new BoNT due to conjugative plasmid transfer could make the strain multivalent, leading to difficulties in detection, diagnosis, and treatment. Conversely, experimental conjugation of plasmid-borne toxins to heterologous hosts (e.g. Fig. 5) could aid in BoNT production and purification.

pCLJ transferred with the highest frequency to *C. botulinum* Hall A-*hyper*, a strain identified for maximal BoNT/A production (Table 2). Hall A-*hyper* is a unique lab-adapted strain; it is missing cell surface and membrane proteins that are present in reference *C. botulinum* genomes^39^. The loss of these features may allow closer cell-cell contact between mating pairs, perhaps enabling pCLJ to transfer more easily to Hall A-*hyper* recipients. Physical barriers are only one impediment to conjugation between bacterial strains, however, and there must surely be other factors that contribute to the observed discrepancies in transfer frequency across the clostridia. The genetic diversity between donor and recipient strains did not show a clear correlation to the transfer frequency within the mating pair (Table 2).

In all transconjugants we monitored, pCLJ was maintained in at least one full-length extrachromosomal copy (Figs 3 and 4). In two transconjugants, we observed the appearance of a larger XhoI-generated fragment that hybridized with *bont/a4* probes (Fig. 4). Further analysis is necessary to ascertain whether this signal is due to recombination of the plasmid-borne *bont/a4* gene with a chromosomal *bont* gene, incomplete restriction digest of the sample, altered plasmid mobility, or another factor. In the vast majority of transconjugants, including all *C. sporogenes* transconjugants, we did not observe the integration or recombination of the *bont/a4* gene between plasmid and chromosomal replicons. Intriguingly, sequence information from a newly identified BoNT/FA-producing strain suggests that toxin genes may have moved from plasmid to chromosome or the reverse. This mosaic toxin is encoded in a unique chromosomal locus compared to other *bont* genes, is adjacent to a “pCLJ-like” region, and is flanked by IS elements, implying that the toxin cluster was moved between replicons by an unknown recombination event^40^.

In gram-positive bacteria, conjugative transfer systems often bear a resemblance to type IV secretion systems, including ATPases, mating channel proteins, and coupling proteins^41^. In *Clostridium*, the most well-characterized conjugation system is the *tcp* locus of *C. perfringens* toxin plasmids^22^. These are typified by pCW3, a ~47 kb plasmid that confers tetracycline resistance and transfers at high frequency. Intriguingly, the *tcp* locus is similar to the conjugation locus from Tn*916*^42^. The pCLJ sequence annotations do not indicate any obvious parallels to the pCW3/Tn*916* model, and accordingly the transfer frequencies reported here are several orders of magnitude lower than observed in *C. perfringens*^43^. There is, however, a large region of pCLJ that contains many type II/IV secretion components in addition to an ATPase, a helicase, and a cell wall hydrolase^44^. A homologous region is seen in the sequence of pCLK, a Group I *C. botulinum* plasmid close in size to pCLJ, which was also experimentally mobilized^32^. The small second plasmid present in the *C. botulinum* 657Ba reference strain (NCBI Assembly GCA_000020345.1) does not contain this so-called “conjugation region” and has very little sequence homology to other clostridial plasmids, as determined via nucleotide BLAST^45^. Still, it is possible that the smaller plasmid and/or genes from the donor chromosome play an accessory role in pCLJ transfer. Efforts are ongoing to determine the functions of genes in the conjugation region of pCLJ and explore their contributions to plasmid transfer in *C. botulinum*. Detailed study of the pCLJ conjugative apparatus may ultimately reveal a much-needed source of genetic tools for molecular manipulation of *C. botulinum*.

This work could also contribute to the enigmatic functions of BoNTs in nature. The neuromuscular junction has been considered the most vulnerable target of toxins leading to morbidity and mortality, and the phrenic nerve controlling breathing is among the most sensitive neuromuscular sites^2^. Considerable work has been performed on the microbe-mediated decomposition of animal and human carcasses^46,47^, but these studies have been done on animals following their death. The actual events that trigger deaths in animals are not always well-defined, but botulinum toxins are known to cause hundreds of thousands of deaths in wildlife every year^48^, and it is plausible that botulinum toxins play such a role in death and ensuing animal decomposition and recycling of nutrients.

## Methods

### Biosafety and biosecurity

*Clostridium botulinum* and BoNTs (>1 mg) are classified as Tier 1 Category A Select Agents, the highest security group of biological agents. The Johnson laboratory and personnel are registered with the Federal Select Agent Program for research involving BoNTs and BoNT-producing strains of clostridia. The research program, procedures, documentation, security, and facilities are closely monitored by the University of Wisconsin-Madison Biosecurity Task Force, University of Wisconsin-Madison Office of Biological Safety, University of Wisconsin Select Agent Program, and the Centers for Disease Control and Prevention. All personnel continually undergo suitability assessments and rigorous biosafety training, including biosafety level 3 (BSL3) or BSL2 and select agent practices, before participating in laboratory studies involving BoNTs and neurotoxigenic *C. botulinum*. All plasmid transfer experiments were conducted using plasmid pCLJ on which the major *bont* gene had been genetically inactivated by ClosTron^32^. The second plasmid-borne *bont* gene produces the minor toxin BoNT/A4, which has an LD_50_ greater than 100 ng/kg^49,50^. As the toxicity is above this threshold, creating new strains of bacteria that express BoNT/A4 is not designated as a restricted experiment/major action according to the Federal Select Agent Regulations and the *NIH Guidelines*.

### Bacterial strains and culture conditions

Clostridial strains were maintained at 37 °C on the following media: TPGY (50 g/l trypticase peptone, 5 g/l Bacto peptone, 4 g/l dextrose, 20 g/l yeast extract, 1 g/l cysteine-HCl, pH 7.4), TPM (20 g/l casein hydrolysate [NZ Case TT; Kerry Bio-Science, Beloit, WI], 10 g/l yeast extract, 5 g/l glucose, pH 7.2), TYG (rich medium; 30 g/l Bacto tryptone, 20 g/l yeast extract, 1 g/l sodium thioglycolate), and MI (minimal medium)^36^. Auxotrophs were screened on MI with the addition of 2 g/l lysine. Solid media for mating contained 4% agar; all other plates were prepared with 1.5% agar. Frozen stocks consisted of TPGY cultures supplemented with 20% glycerol and stored at −80 °C. Erythromycin was used at 50 μg/ml where appropriate. All chemicals and media components were purchased from Becton Dickinson Microbiology Systems (Sparks, MD) and Sigma-Aldrich (St. Louis, MO) unless otherwise noted.

Cultures were grown under anaerobic conditions. Glass culture tubes were flushed with nitrogen gas and sealed with butyl rubber stoppers (Bellco Glass, Vineland, NJ) before sterilizing. All culture manipulations were performed in an anaerobic chamber (Forma Anaerobic System, Marietta, OH) with an initial gas mixture of 80% N_2_, 10% CO_2_, and 10% H_2_. Resazurin was added to solid media at 2 μg/ml, and agar plates were prereduced by overnight incubation in the anaerobic chamber.

### Creation of an auxotrophic plasmid donor strain

*C. botulinum* strain 657Ba, containing the dual-BoNT-encoding plasmid pCLJ, served as the donor strain in this study. Prior work in our laboratory utilized ClosTron technology^51^ to insertionally inactivate the *bont/b* gene on pCLJ, conferring erythromycin resistance on the strain^32^. The Em^R^ parent strain was subsequently chemically mutagenized with 1 mg/ml *N*-methyl-*N*′-nitro-*N*-nitrosoguanidine (MNNG; TCI America, Portland, OR) in a protocol adapted from the literature^52,53^. Cultures were revived from frozen stock, subcultured once, and grown to mid-log phase. Cells were collected by centrifugation, washed once with citrate buffer (100 mM citric acid – sodium citrate, pH 5.5) and resuspended in the same buffer. MNNG in citrate buffer was added to achieve a final concentration of 1 mg/ml. The cells were incubated in a 37 °C water bath for 15 minutes. MNNG was then quenched with the addition of 0.5 ml cold sodium phosphate buffer (100 mM, pH 7). Cells were harvested by centrifugation, washed twice with phosphate buffer, and resuspended in 0.5 ml buffer. The entire volume was then added to fresh TPGY broth and allowed to outgrow overnight at 37 °C. Cultures were diluted and plated on TYG agar for isolated colonies. Colonies that grew on rich medium were picked with sterile toothpicks and replica plated on minimal media to identify auxotrophs. An isolate that grew on TYG and MI + lysine but failed to grow on MI was restreaked for purification and chosen for further analysis. After confirming that this isolate retained pCLJ, remained resistant to erythromycin, and suffered no growth defects in rich medium, clonal isolates of the mutant strain were utilized as a plasmid donor in all mating experiments described herein (EMN053-EMN056, Table 1).

### Plasmid transfer

Donor and recipient strains were revived from frozen stock, subcultured twice, and grown to OD_600_ ≈ 0.8. In order to enumerate donors and recipients, 200 μl of each culture was spotted individually on TYG with 4% agar. To assess plasmid transfer, equal volumes (200 μl) of donor and recipient were spotted together on TYG with 4% agar. All agar plates were allowed to incubate at 37 °C for 18 h. Bacterial growth was then washed with 3 ml 1× PBS, and the suspensions were serially diluted in 1× PBS. Dilutions of donor and recipient controls were plated on TYG with erythromycin and on MI, respectively, to determine CFU/ml. Aliquots of donor suspensions (100 μl) were spread on minimal media to test for reversion to prototrophy. Likewise, aliquots of recipient suspensions (100 μl) were spread on TYG with erythromycin to account for spontaneous mutation. Mating mixtures were diluted and plated on MI supplemented with erythromycin to enumerate transconjugants. Transfer frequency was calculated by dividing transconjugant CFU/ml by the larger of the donor or recipient CFU/ml.

### Analysis of transconjugant strains

In order to verify that the observed colonies were true transconjugants (recipient strains that obtained the donor plasmid), a variety of screens were performed. Spontaneous Em^R^ mutants were exceedingly uncommon, and recipient strains were often visually different from the plasmid donor. Thus, colony morphology on MI + Em was typically sufficient to differentiate transconjugants from revertants. For mating pairs in which donor and recipient strains were morphologically similar, a marker gene characteristic of the recipient was amplified by PCR. Putative transconjugant colonies were picked with sterile toothpicks, swirled in 200 μl sterile water, and microwaved for 1 minute. One microliter of this template DNA was dispensed directly into *Taq* 2X Master Mixes (New England Biolabs, Ipswich, MA) and marker genes amplified using appropriate primers (Table 1).

The presence of pCLJ was also confirmed in representative transconjugants by pulsed-field gel electrophoresis (PFGE). Strains were revived from frozen stock, grown anaerobically overnight at 37 °C, and subcultured into 10 ml TPGY broth. Growth of the subcultures was then monitored until their OD_600_ reached 1.2 (ThermoFisher Spectronic 200, Waltham, MA), at which point 1 ml formaldehyde was added and the cultures were placed on ice. PFGE plugs were prepared as previously described^54^. One plug from each sample was processed without restriction enzyme digest, and a second was digested with XhoI (New England Biolabs, Ipswich, MA), a rare cutting enzyme that linearizes pCLJ. DNA was separated by electrophoresis in a clamped homogeneous electric field system at 6 V/cm, 12 °C, 1–26 s pulse time for 24 h (CHEF-DRII; Bio-Rad, Hercules, CA). Samples were stained with 1 μg/ml ethidium bromide for 20 min, washed in sterile water 5 × 30 min to destain, and photographed using a Fotodyne/FOTO/Analyst FX imaging system and software (Harland, WI). If necessary, image brightness and contrast were equally adjusted using Adobe Photoshop CC software.

To prepare PFGE samples for Southern hybridization, gels were transferred to a Nytran SuPerCharge membrane (GE Healthcare Life Sciences, Marlborough, MA) overnight by downward capillary transfer^55^. Membranes were wet in distilled water for 15 min, washed in transfer buffer (0.4 M NaOH, 1.5 M NaCl) for 15 min, and transferred using the GE Healthcare Life Sciences Turboblotter stack (Marlborough, MA) for approximately 16 h. Following transfer, the membranes were neutralized for 15 min in 2 M Tris-HCl, pH 7.0. They were then rinsed for 15 min in 2× SSC (3 M NaCl, 0.3 M sodium citrate) and dried under vacuum for 1 h at 80 °C. Membranes were stored at 4 °C until hybridization. Hybridization probes were amplified from a recombinant plasmid copy of *bont/a4*^49^ by PCR with Phusion High Fidelity Master Mix (New England Biolabs, Ipswich, MA) using the three A4 primer pairs listed in Table 1. Gene fragments were purified from agarose gels using Qiagen’s MinElute kit (Germantown, MD) and radiolabeled with α-^32^P ATP using the Megaprime DNA labeling system (GE Healthcare Life Sciences, Marlborough, MA). Membranes were individually pre-washed in a solution of 50 mM Tris-HCl pH 8.0, 1 M NaCl, 1 mM EDTA, and 0.1% SDS for 2.5 h at 60 °C, then rinsed with 5× SSPE. Each membrane was incubated for 2.5 h at 42 °C in 6× Denhardt’s solution, 5× SSPE, 50% formamide, 0.5% SDS, and 100 μg/ml herring sperm DNA (Promega, Madison, WI). A mixture of the three radiolabeled probes was then added to each bottle at approximately 5 × 10^7^ cpm/ml and hybridization carried out for 17 h at 42 °C. Membranes were washed (a) twice with 1× SSPE and 0.1% SDS for 5 min at RT, (b) once with 1× SSPE and 0.1% SDS for 10 min at 42 °C, and (c) once with 0.1× SSPE and 0.1% SDS for 10 min at 42 °C. Autoradiography took place at −80 °C overnight using blue x-ray film (Phenix Research Products, Candler, NC) and a BioMax intensifying screen (Eastman Kodak, Rochester, NY).

Transconjugant strains were analyzed at the protein level by SDS-PAGE and Western blotting. Strains were revived from frozen stock and cultured in TPM at 37 °C for 48 h. Subsamples of the whole cultures were removed anaerobically and centrifuged 5 min to pellet cells. A portion of the supernatant was treated with 50 μg/ml TPCK-treated trypsin (Worthington Biochemical Corp., Lakewood, NJ) for 30 min at 35 °C. Type II-S soybean trypsin inhibitor (Sigma-Aldrich, St. Louis, MO) was then added to this portion at a final concentration of 100 μg/ml and incubated at room temperature for 10 min. All samples were denatured by adding NuPAGE SDS sample buffer (Life Technologies, Carlsbad, CA) to 1× concentration and heated to 95 °C for 5 minutes. Samples were reduced with the addition of 100 mM DTT as desired. Proteins were separated by electrophoresis at 150 V through 4–12% Bis-Tris NuPAGE Novex gels in morpholineethane sulfonic acid (MES) running buffer (Life Technologies, Carlsbad, CA). The gels were stained using Instant Blue (Expedeon Inc., San Diego, CA), and various markers including the SeeBlue Plus2 Prestained Standard and the MagicMark XP Western Standard (Life Technologies, Carlsbad, CA) were used to estimate molecular weight. Gels were transferred to PVDF membranes (MilliporeSigma, Burlington, MA) using a semi-dry transfer protocol (0.4 A, 1 h). Membranes were probed for the presence of BoNT/A with polyclonal affinity-purified rabbit IgG antibodies raised in our laboratory against BoNT/A1 and a bovine anti-rabbit secondary antibody (Santa Cruz Biotechnology, Dallas, TX). Images were developed with the chemiluminescent PhosphaGLO alkaline phosphatase substrate (KPL, Gaithersburg, MD) and photographed as above.

### Mouse bioassay

Toxicity of culture supernatants was determined by mouse bioassay as previously described^56,57^. Briefly, culture supernatants from parent strains and transconjugant derivatives grown at 37 °C for 48 h were passed through a 0.2 μm filter. To evaluate neutralization, monovalent antitoxin produced in our lab was incubated with culture supernatant at ambient temperature for 60 min. All samples were diluted three-fold in 0.03 M sodium phosphate buffer (pH 6.3) with 0.2% gelatin (GelPhos). Two mice per sample were injected intraperitoneally with 0.5 ml of the GelPhos dilution (i.e. approximately 0.167 ml of the culture supernatant) and observed for signs of botulism for 4 days^58^. The mouse research was conducted according to protocols approved by the CDC Institutional Animal Care and Use Committee.

### Statistical analysis

Selected mating assays were compared using an unpaired Student’s *t*-test. The difference in transfer frequencies between two recipient strains is described as statistically significant if *P* ≤ 0.05.

## Electronic supplementary material

Supplementary Information
