# Supplementary material for: Botulinum neurotoxin–encoding plasmids can be conjugatively transferred to diverse clostridial strains
Source: Sci Rep. 2018 Feb 15;8:3100. doi: 10.1038/s41598-018-21342-9 (PMC5814558; doi:10.1038/s41598-018-21342-9)
Supplement: Supplementary file 1 — Supplementary Information [file 41598_2018_21342_MOESM1_ESM.pdf]

Supplementary Information

Botulinum neurotoxin–encoding plasmids can be conjugatively transferred to diverse clostridial strains

Erin M. Nawrocki, Marite Bradshaw, Eric A. Johnson*

Department of Bacteriology, University of Wisconsin-Madison, Madison, Wisconsin, USA

*Address correspondence to eric.johnson@wisc.edu

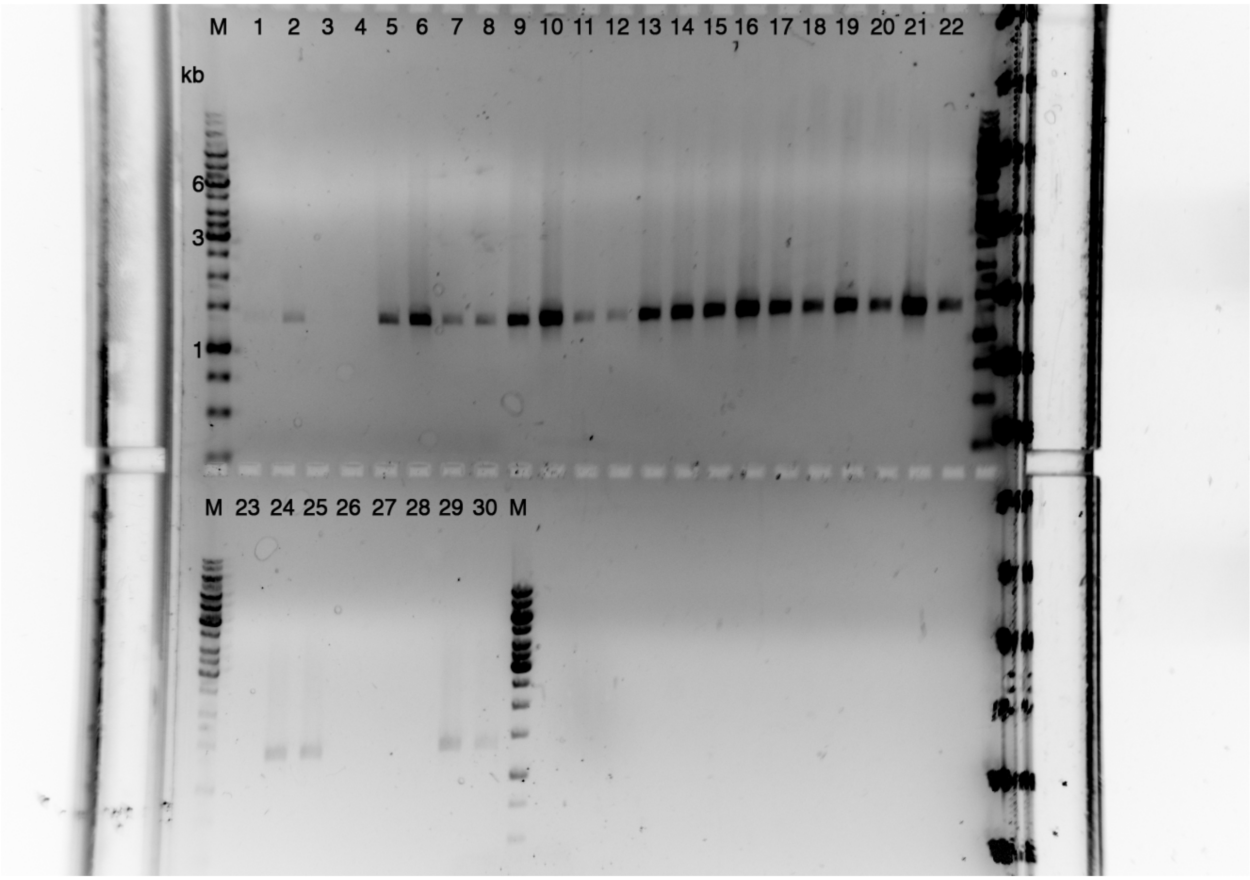

Supplementary Figure S1. Colony PCR confirms transconjugant genotype. A marker gene characteristic of the recipient strain (*bont/a1*) was amplified by colony PCR using the A1 primer pair in Table 1 and analyzed by agarose gel electrophoresis. Lanes 1-2, recipient strains; lanes 3-4, pCLJ donor strains; lanes 5-22, selected transconjugants. M, O'GeneRuler 1 kb DNA ladder (Thermo Scientific, Waltham, MA). Lanes 23-30 show a colony PCR screen from an independent conjugation experiment.

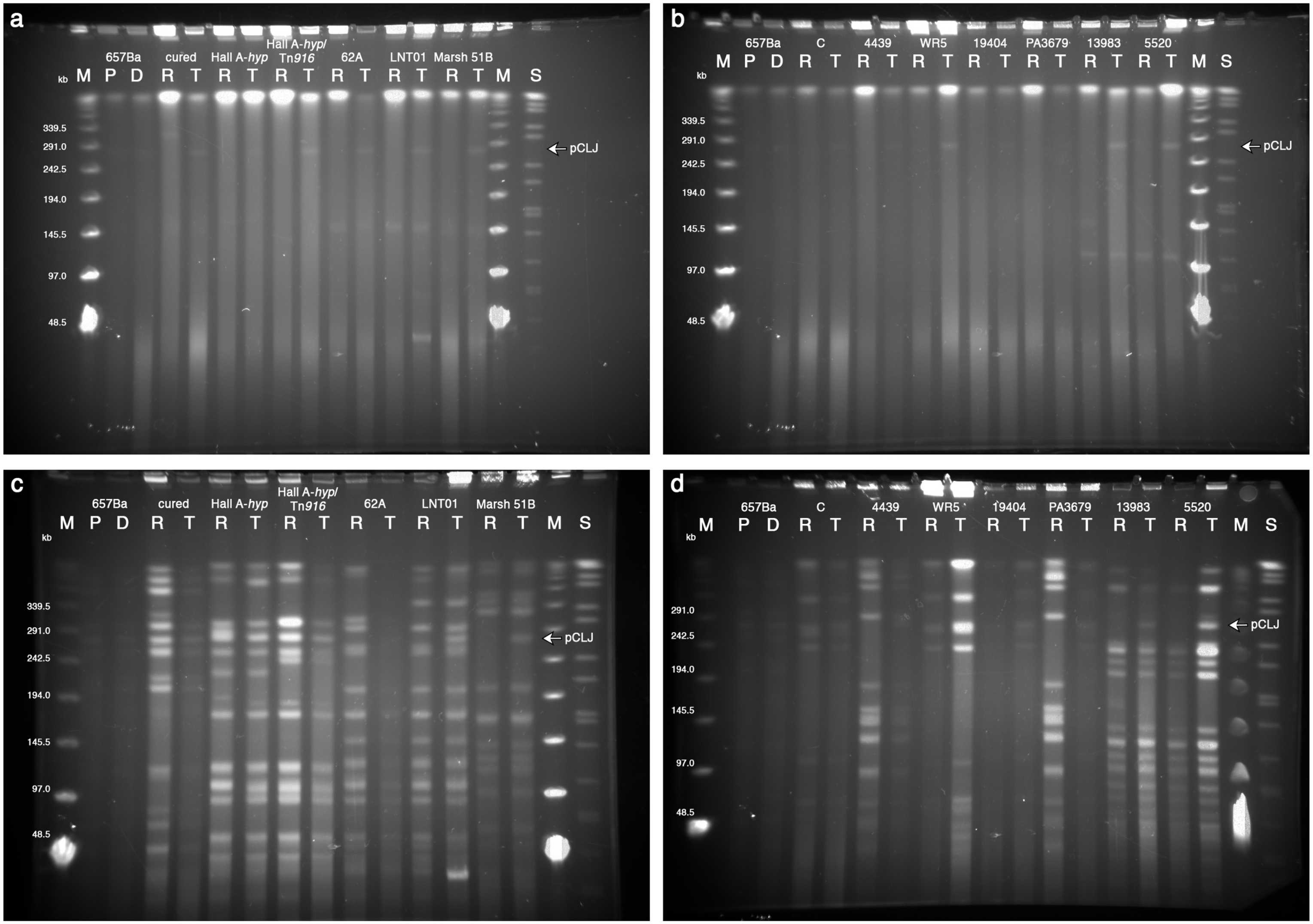

Supplementary Figure S2. Full-length pCLJ is maintained in transconjugant strains. Matings were conducted as described in the text. PFGE plugs were prepared from the given strains and electrophoresed undigested (a-b) and following XhoI digest (c-d). pCLJ, at ~270 kb, is marked with an arrow. P, *C. botulinum* 657Ba (pCLJ-Erm) parent strain; D, pCLJ auxotrophic donor strain; R, recipient strains; T, transconjugant strains; M, Lambda PFG Ladder; S, *Salmonella enterica* serotype Braenderup strain H9812, XbaI digest.

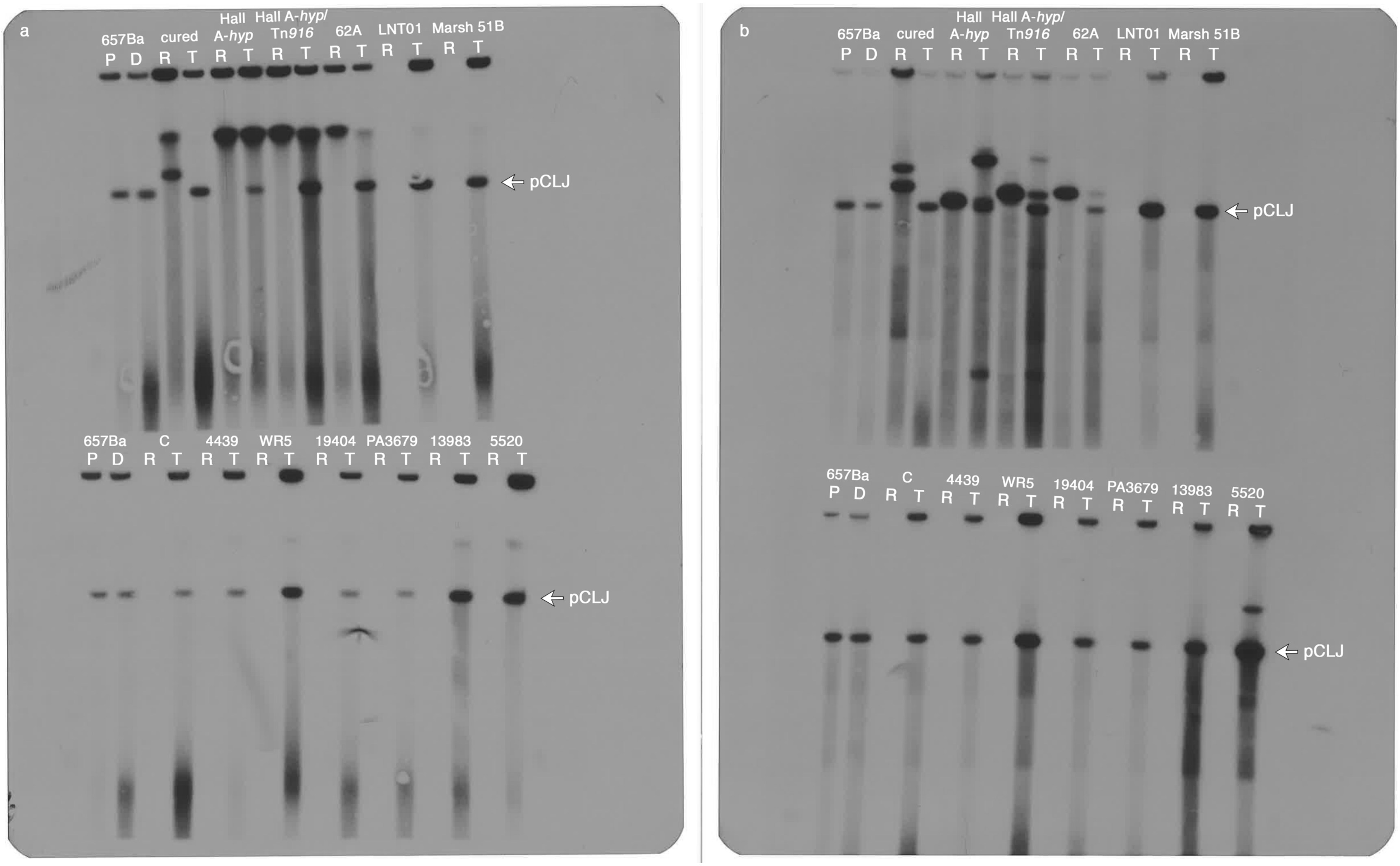

Supplementary Figure S3. ***bont/a4*** **remains associated with pCLJ in transconjugant strains.** Undigested PFGE samples (a) and XhoI-digested samples (b) were transferred to nylon membranes and hybridized to *bont/a4* probes. pCLJ, at ~270 kb, is marked with an arrow. P, *C. botulinum* 657Ba (pCLJ-Erm) parent strain; D, pCLJ auxotrophic donor strain; R, recipient strains; T, transconjugant strains.

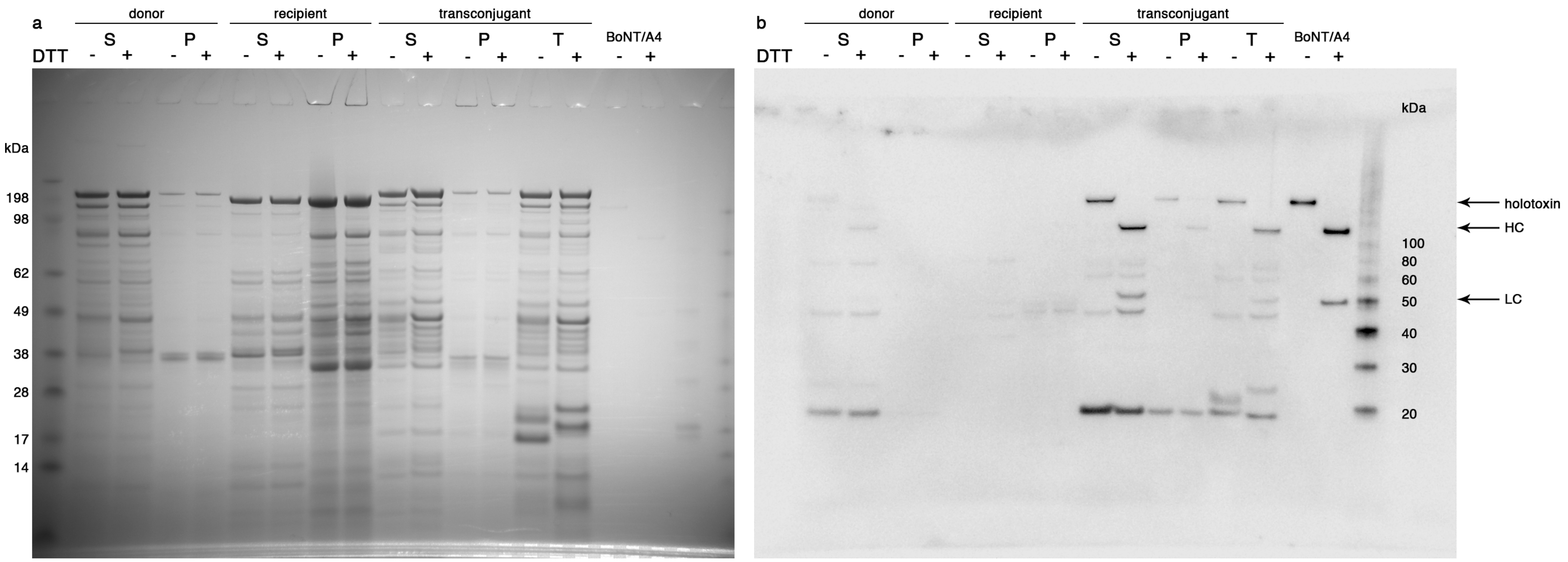

Supplementary Figure S4. Transconjugant strains are converted to toxigenicity. Samples of donor strain EMN053, recipient strain *C. sporogenes* PA3679, and transconjugant strain *C. sporogenes* PA3679 (pCLJ) were collected at 48 h and prepared as described in the text. S, supernatant; P, pellet; T, trypsinized supernatant. Gels were (a) stained to visualize total proteins or (b) immunoblotted for the presence of BoNT/A. Purified BoNT/A4 was used as a control. Samples were reduced with 100 mM DTT as indicated.

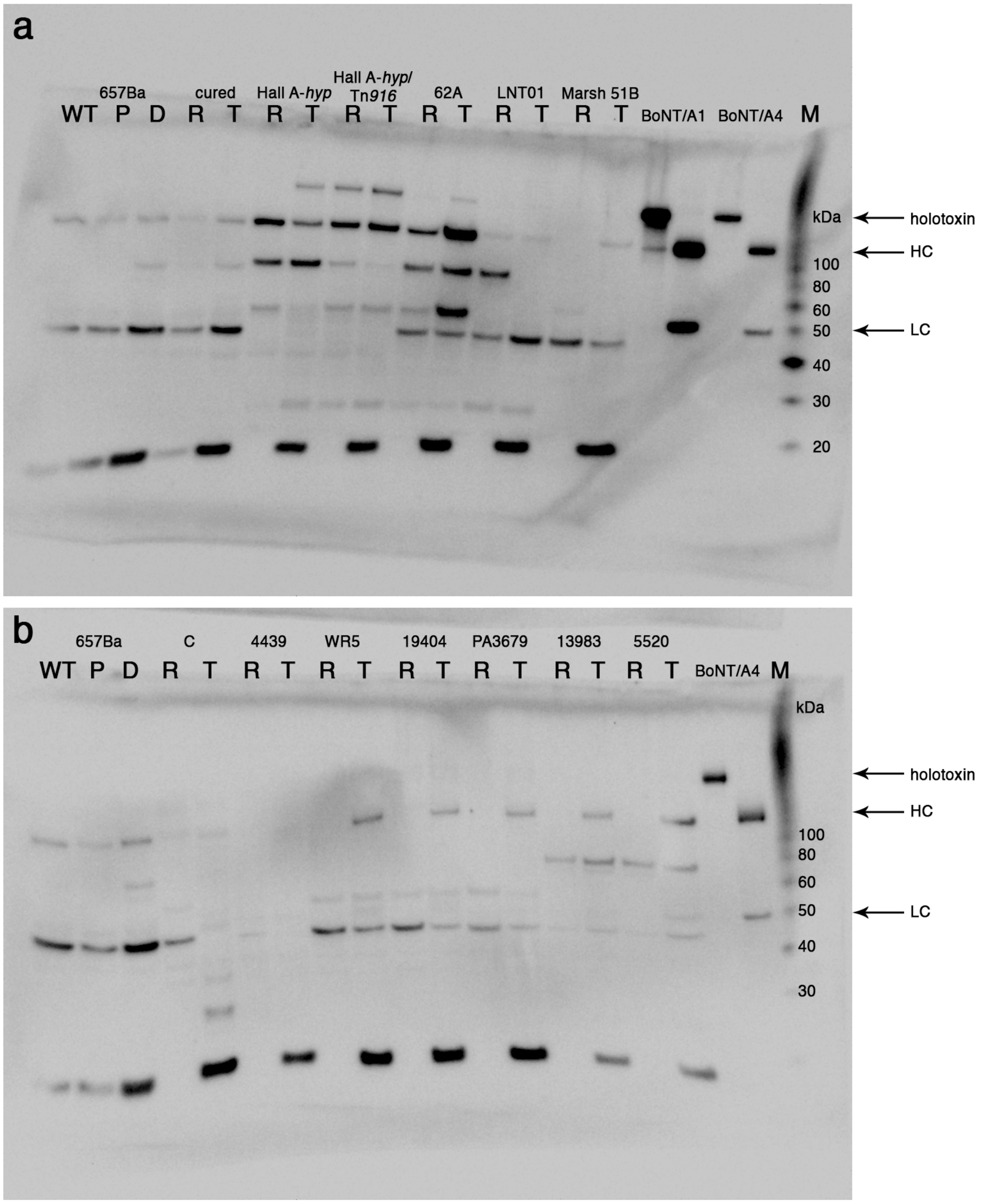

Supplementary Figure S5. Transconjugant strains can be identified by immunoblot. Representative donor, recipient, and transconjugant strains were grown for 96 h in TPGY and sampled and analyzed as described in the text. All culture lysates were reduced with 100 mM DTT; the BoNT/A toxin controls were divided and only one portion, at right, was reduced. WT, *C. botulinum* 657Ba wild-type; P, *C. botulinum* 657Ba (pCLJ-Erm) parent strain; D, auxotrophic donor; R, recipient; T, transconjugant; M, Magic Mark XP Western Protein Standard; HC, heavy chain; LC, light chain.
